# CD163 as a Biomarker in Colorectal Cancer: The Expression on Circulating Monocytes and Tumor-Associated Macrophages, and the Soluble Form in the Blood

**DOI:** 10.3390/ijms21165925

**Published:** 2020-08-18

**Authors:** Daniëlle Krijgsman, Natasja L. De Vries, Morten N. Andersen, Anni Skovbo, Rob A.E.M. Tollenaar, Holger J. Møller, Marianne Hokland, Peter J.K. Kuppen

**Affiliations:** 1 Department of Surgery, Leiden University Medical Center, P.O. Box 9600, 2300 RC Leiden, The Netherlands; d.krijgsman@lumc.nl (D.K.); N.L.de_Vries@lumc.nl (N.L.D.V.); R.A.E.M.Tollenaar@lumc.nl (R.A.E.M.T.); 2 Department of Biomedicine, Aarhus University, 8000 Aarhus, Denmark; morten@biomed.au.dk (M.N.A.); anniskovbo@biomed.au.dk (A.S.); mhokland@biomed.au.dk (M.H.); 3 Department of Clinical Biochemistry, Aarhus University Hospital, 8200 Aarhus, Denmark; holgmoel@rm.dk; 4 FACS Core Facility, Aarhus University, 8200 Aarhus, Denmark

**Keywords:** colorectal cancer, prognostic immune markers, monocytes, tumor-associated macrophages, soluble CD163, regulatory T cells

## Abstract

The macrophage-associated molecule CD163 has been reported as a prognostic biomarker in different cancer types, but its role in colorectal cancer (CRC) is unclear. We studied CD163 in the tumor microenvironment and circulation of patients with CRC in relation to clinicopathological parameters. An enzyme-linked immunosorbent assay (ELISA) was used to measure the serum sCD163 levels and multiparameter flow cytometry was used to study the peripheral blood monocytes and their CD163 expression in CRC patients (*N* = 78) and healthy donors (*N* = 50). The distribution of tumor-associated macrophages (TAMs) was studied in primary colorectal tumors with multiplex immunofluorescence. We showed that CRC patients with above-median sCD163 level had a shorter overall survival (OS, *p* = 0.035) as well as disease-free survival (DFS, *p* = 0.005). The above-median sCD163 remained significantly associated with a shorter DFS in the multivariate analysis (*p* = 0.049). Moreover, a shorter OS was observed in CRC patients with an above-median total monocyte percentage (*p* = 0.007). The number and phenotype of the stromal and intraepithelial TAMs in colorectal tumors were not associated with clinical outcome. In conclusion, sCD163 and monocytes in the circulation may be potential prognostic biomarkers in CRC patients, whereas TAMs in the tumor showed no association with clinical outcome. Thus, our results emphasize the importance of the innate systemic immune response in CRC disease progression.

## 1. Introduction

Colorectal cancer (CRC) remains one of the leading causes of cancer-related deaths worldwide [1]. Approximately 25% of CRC patients have distant metastases at diagnosis [2]. Additionally, up to 25% of the patients diagnosed in the early stages eventually relapse or develop distant metastases following radical surgery and adjuvant chemotherapy [2,3]. In order to optimize treatment strategies, it is crucial that biomarkers are identified that associate with clinical outcome. Due to its critical role in combating tumor development and progression, the immune system has become an important focus in biomarker research. Studies have indicated important roles for monocytes and macrophages in CRC development and progression [4].

Monocytes can be divided into subsets based on their CD14 and CD16 expression levels. Classical monocytes (CD14^++^CD16^−^) develop in the bone marrow from myeloid progenitor cells and enter the circulation where they may differentiate into intermediate monocytes (CD14^++^CD16^+^) and, subsequently, to nonclassical monocytes (CD14^+^CD16^++^) [5]. Classical monocytes are the most prevalent subset in peripheral blood and are important phagocytes [6]. Intermediate monocytes are potent producers of pro-inflammatory cytokines, whereas nonclassical monocytes produce anti-inflammatory cytokines [6]. Recent meta-analyses have shown that, considering peripheral blood leukocytes, a high lymphocyte-to-monocyte ratio was a significant predictor of better overall survival (OS), disease-free survival (DFS) and cancer-specific survival in CRC patients [7,8]. However, circulating monocyte subsets and the monocyte–macrophage marker CD163 have not been widely investigated in CRC patients.

CD163 is a 130-kDa transmembrane scavenger receptor solely expressed by monocytes and macrophages mediating the endocytic uptake of haptoglobin–hemoglobin (Hp-Hb) complexes that form upon intravascular hemolysis [9]. Upon internalization, Hp-Hb complexes are degraded in lysosomes thereby producing anti-inflammatory heme metabolites [9] that dampen the inflammatory response of monocytes and macrophages [10]. CD163 can be cleaved from the cell membrane of monocytes and macrophages by the protease ADAM17/TACE upon activation by pro-inflammatory stimuli [11]. Soluble CD163 (sCD163) is an important biomarker in various inflammatory diseases including sepsis, liver disease, and macrophage activation syndrome [12]. In addition, high sCD163 levels have been associated with disease progression and clinical outcome in different cancer types [13,14,15,16,17].

When monocytes leave the circulation and migrate into tissue, they differentiate into macrophages. Uncommitted M0 macrophages have been described to polarize into pro-inflammatory macrophages (the so-called M1 phenotype) with a high inducible nitric oxide synthase (iNOS) expression, or into macrophages associated with wound healing and anti-inflammatory functions (the so-called M2 phenotype) with a high CD163 expression [18,19]. Tumor-associated macrophages (TAMs) have been reported to express high levels of CD163 (i.e., M2 phenotype) and the density of these TAMs is associated with unfavorable clinical outcome in numerous human cancers [20,21,22]. Additionally, M3 TAMs have also been described with an M1/M2 or M2/M1 switch phenotype, both in mice [23] and humans [24,25].

Although CD163 has been reported a prognostic biomarker in different cancer types, its role in CRC is still unclear and requires further investigation. For instance, a high CD163^+^ TAM density has been reported to associate with both unfavorable [26,27,28] and favorable clinical outcome [24,25,29,30,31] in CRC. Therefore, we decided to study CD163 in a broader context, comprising both the tumor microenvironment and circulation of CRC patients. We investigated CD163 expressed by circulating monocytes and TAMs, and the sCD163 in the blood in relation to clinicopathological parameters in CRC.

## 2. Results

### 2.1. Study Population

**Figure 1:**
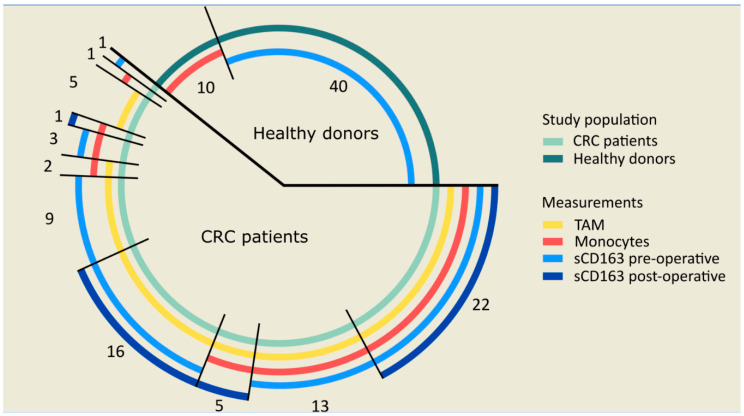
Sample availability for the measurement of monocytes, sCD163 and tumor-associated macrophages (TAMs) in colorectal cancer (CRC) patients and healthy donors. Monocytes, sCD163 and TAMs were studied in 78 CRC patients. TAMs and monocytes were studied in 72 and 47 CRC patients, respectively. Additionally, sCD163 levels were studied in 64 pre-operative and 44 post-operative patients. Finally, monocytes were studied in 10 healthy donors, whereas sCD163 levels were studied in 40 healthy donors. The numbers in the figure indicate the number of patients in each subgroup with overlapping samples. Abbreviations: CRC (colorectal cancer), ELISA (enzyme-linked immunosorbent assay), PBMC (peripheral blood mononuclear cells), sCD163 (soluble CD163), TAM (tumor-associated macrophages).

We investigated CD163 expressed by circulating monocytes and TAMs, and its soluble circulating form (sCD163) in relation to the clinicopathological parameters in CRC. In total, 78 CRC patients were included in the study. Due to a limited sample availability, sCD163, monocytes, and TAMs were studied in subgroups of this cohort, as visualized in Figure 1. As controls, sCD163 was studied in the serum of 40 healthy donors. Additionally, the CD163 expression on circulating monocytes was studied in 10 healthy donors. The clinicopathological characteristics of the 78 CRC patients and healthy donors are summarized in Table 1. No differences were observed between the distribution of age or sex between the 78 CRC patients and the 40 healthy serum donors. The age of the healthy PBMC donors was significantly lower than the CRC patients (*p* = 0.028). This was due to the limited PBMC sample availability from elderly healthy donors. No differences were found regarding the distribution of sex between patients and healthy PBMC donors.

### 2.2. Trend towards Increased sCD163 Levels in CRC Patients with a Higher TNM Classification

**Figure 2:**
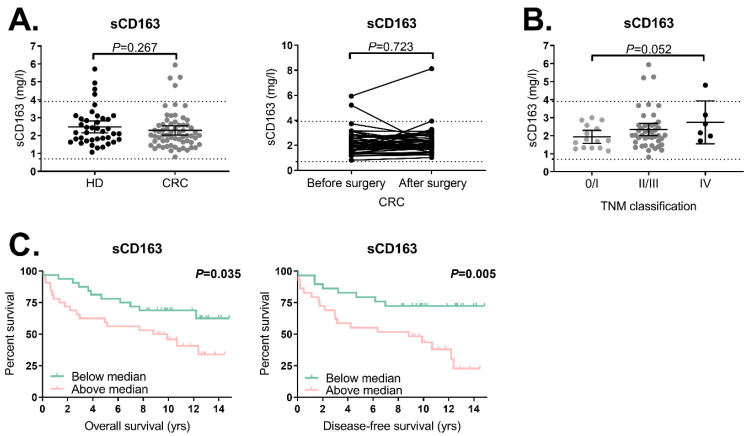
sCD163 levels in the serum of CRC patients and healthy donors as measured by the enzyme-linked immunosorbent assay (ELISA) in relation to clinicopathological parameters. (**A**) Comparison of sCD163 serum levels in healthy donors (*N* = 40) and pre-operative CRC patients (*N* = 64), and the change in sCD163 levels in CRC patients after surgery (*N* = 39). (**B**) Association between the sCD163 levels in CRC patients and TNM stage (stage 0/I, *N* = 15; stage II/III, *N* = 43; stage IV, *N* = 6). (**C**) Association between the sCD163 levels and clinical outcome in CRC patients. Kaplan–Meier curves for the overall survival (OS) are shown for TNM stage 0–IV CRC patients (*N* = 64) and Kaplan–Meier curves for disease-free survival (DFS) are shown for the TNM stage 0–III CRC patients (N = 58). Stratifications were based on the median sCD163 level (2.0 mg/L). The bars ((**A**), left figure; (**B**)) show the median sCD163 level with a 95% confidence interval (CI) whereas the dotted lines show the reference sCD163 levels (0.7–3.9 mg/L). Statistically significant *p*-values (≤0.05) are indicated in bold. Abbreviations: CI (confidence interval), CRC (colorectal cancer), DFS (disease-free survival), HD (healthy donor), OS (overall survival), sCD163 (soluble CD163), TNM (tumor, node, metastasis).

We studied the levels of sCD163 in the pre-operative (*N* = 64) and post-operative serum samples (*N* = 44) derived from CRC patients and in 40 healthy donors. The majority of the measured sCD163 levels from healthy donors and CRC patients were within the reference range (0.7–3.9 mg/L) with no difference in the sCD163 levels between the two groups (Figure 2A, *p* = 0.267). In the 39 patients with pre-operative and post-operative serum samples available, we observed that sCD163 levels did not change after resection of the tumor (*p* = 0.723, Figure 2A). We also investigated the association between sCD163 levels and tumor characteristics (Table S1A). Although no correlation was observed between the sCD163 levels and TNM stage in a Spearman’s rho correlation test (*p* = 0.141), an intergroup analysis revealed that patients with TNM stage IV tumors showed a trend towards higher sCD163 levels compared to TNM stage 0/I patients (*p* = 0.052, Figure 2B). No association was observed between the sCD163 levels in CRC patients and tumor location, differentiation grade or tumor–lymph node invasion (Table S1A).

### 2.3. High sCD163 Levels Are Associated with a Shorter OS and DFS in CRC Patients

Next, the association between the sCD163 levels and clinical outcome was investigated in CRC patients. The patient population (*N* = 64) was divided into two groups using the median concentration of sCD163 (2.0 mg/L) as a cutoff. We observed that above-median sCD163 levels in CRC patients were associated with a shorter OS (*p* = 0.035), with a hazard ratio (HR) of 2.2 (95% confidence interval (CI) 1.0–4.6, *p* = 0.040). Patients with TNM stage IV tumors were excluded from the DFS analyses (*N* = 6) since they already presented metastatic disease at the time of blood sampling. Patients with above-median sCD163 levels showed a significantly shorter DFS (*p* = 0.005) compared to patients with below-median sCD163 levels, with a hazard ratio (HR) of 3.1 (CI 1.4–7.1, *p* = 0.007) (Figure 2C). A multivariate analysis was performed for DFS and OS in CRC patients which revealed that above-median sCD163 levels (HR 2.4, 95% CI 1.0–5.7, *p* = 0.049) remained significantly associated with a shorter DFS when corrected for age (category ≤70 or >70 years) and TNM classification (Table 2), but not with the OS (HR 1.5, 95% CI 0.7–3.3, *p* = 0.291, Table 3).

### 2.4. Expression of Membrane-Bound CD163 on Circulating Classical Monocytes Is Decreased in CRC Patients Compared to Healthy Donors

**Figure 3:**
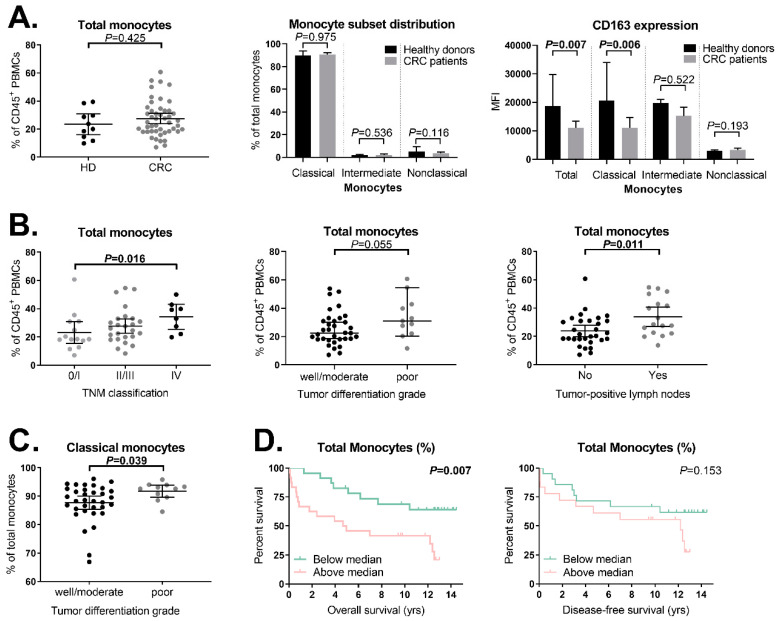
Distribution of monocyte subsets and their level of CD163 expression in the peripheral blood of CRC patients and healthy donors as measured by flow cytometry in relation to clinicopathological parameters (**A**) Comparison of the total monocyte percentage, monocyte subset distribution (CD14^++^CD16^−^ classical, CD14^++^CD16^+^ intermediate and CD14^+^CD16^++^ nonclassical monocytes), and CD163 expression level on these monocyte subsets between healthy donors (*N* = 10) and CRC patients (*N* = 47). (**B**) Association between the total monocyte percentage and TNM stage (stage 0/I, *N* = 14; stage II/III, *N* = 25; stage IV, *N* = 8), differentiation grade (well/moderate, *N* = 34; poor, *N* = 11) and tumor-positive lymph nodes (no, *N* = 30; yes, *N* = 17) in CRC patients. (**C**) Association between the percentage of classical monocytes and differentiation grade (well/moderate, *N* = 34; poor, *N* = 11) in CRC patients. (**D**) Association between the total monocyte percentage and clinical outcome in CRC patients. Kaplan–Meier curves for the OS are shown for TNM stage 0–IV CRC patients (*N* = 47) and Kaplan–Meier curves for the DFS are shown for stage 0–III CRC patients (*N* = 39). Stratifications were based on the median total monocyte percentage (24.9%). The bars in figure (**A**–**C**) show the median with a 95% CI. Statistically significant *p*-values (≤0.05) are indicated in bold. Abbreviations: CI (confidence interval), CRC (colorectal cancer), HD (healthy donor), MFI (median fluorescence intensity), PBMCs (peripheral blood mononuclear cells), TNM (tumor, node, metastasis).

We studied the presence of circulating CD14^+^ and/or CD163^+^ monocytes in pre-operative PBMC samples from CRC patients (*N* = 47) and healthy donors (*N* = 10) with multiparameter flow cytometry using a standardized gating strategy (Figure S1). The total monocyte percentage (% of CD45^+^ PBMCs) was comparable between CRC patients and healthy donors (*p* = 0.425, Figure 3A). The monocyte population was further divided into classical (CD14^++^CD16^−^), intermediate (CD14^++^CD16^+^) and nonclassical (CD14^+^CD16^++^) monocyte subsets. No statistically significant differences were observed in the percentage (of total monocytes) of classical (*p* = 0.975), intermediate (*p* = 0.536), or nonclassical (*p* = 0.116) monocytes when CRC patients were compared to healthy donors (Figure 3A). Interestingly, CD163 was expressed to a lower extent in the total monocyte population in CRC patients compared to healthy donors (*p* = 0.007, Figure 3A). The decreased expression of CD163 was observed only in classical monocytes (*p* = 0.006), and not in intermediate (*p* = 0.522) or nonclassical monocytes (*p* = 0.193, Figure 3A). Interestingly, the percentage of total monocytes positively correlated with the percentage of total Tregs (*p* = 0.019, Figure S2). No association was observed between the CD163 expression on monocytes and the percentage of Tregs in the peripheral blood of CRC patients (*p* = 0.745). Additionally, no significant correlation was observed between the CD163 expression on monocytes and serum sCD163 levels in CRC patients (*p* = 0.482, Figure S3).

### 2.5. Increased Monocyte Percentage in More Advanced Tumors

Next, we examined the association between the total monocyte percentage and monocyte subsets (Table S1B) and their level of CD163 expression (Table S1C) with tumor characteristics. A positive correlation was observed between the total percentage of monocytes and TNM stage in CRC patients (*p* = 0.004). An intergroup analysis revealed that patients with TNM stage IV tumors (*N* = 8) showed a significantly higher total monocyte percentage compared to patients with TNM stage 0/I tumors (*N* = 14, *p* = 0.016, Figure 3B). Additionally, patients with poorly differentiated tumors (*N* = 11) showed a trend towards a higher percentage of circulating monocytes compared to patients with well or moderately differentiated tumors (*N* = 34, *p* = 0.055, Figure 3B). This was restricted to the classical monocytes (*p* = 0.039, Figure 3C). Furthermore, the percentage of total monocytes was higher in patients with tumor-positive lymph nodes (*N* = 17) compared to patients without tumor-positive lymph nodes (*N* = 30, *p* = 0.011, Figure 3B). No significant associations were observed between tumor characteristics, the percentage of intermediate or nonclassical monocytes, or CD163 expression (Table S2B,C).

### 2.6. Association between Total Monocyte Percentage and Clinical Outcome in CRC Patients

We subsequently investigated if the total monocyte percentage in CRC patients was associated with clinical outcome. Kaplan–Meier plots and log-rank tests revealed a shorter OS in CRC patients with an above-median (≥24.9%) total monocyte percentage (*N* = 24) compared to CRC patients with a below-median percentage (*N* = 23, *p* = 0.007, Figure 3D) with a HR of 3.0 (95% CI 3.1–7.1, *p* = 0.010). Patients with TNM stage IV tumors were excluded from the DFS analyses (N = 8). No association was observed between the percentage of total monocytes and DFS (*p* = 0.153, Figure 3D) with a HR of 1.9 (95% CI 0.8–4.8, *p* = 0.161). A multivariate analysis showed that the above-median percentage of circulating monocytes was not independently associated with a shorter OS when corrected for age and TNM classification. The percentage of classical, intermediate, and nonclassical monocytes, and the CD163 expression level on monocytes, were not associated with clinical outcome (data not shown).

### 2.7. TAMs in the Stromal Compartment of Primary Colorectal Tumors Have an M2-Polarized (iNOS^−^CD163^+^) Phenotype whereas in the Epithelium M0- (iNOS^−^CD163^−^) and M1-Polarized (iNOS^+^CD163^−^) Phenotypes Are Predominant

**Figure 4:**
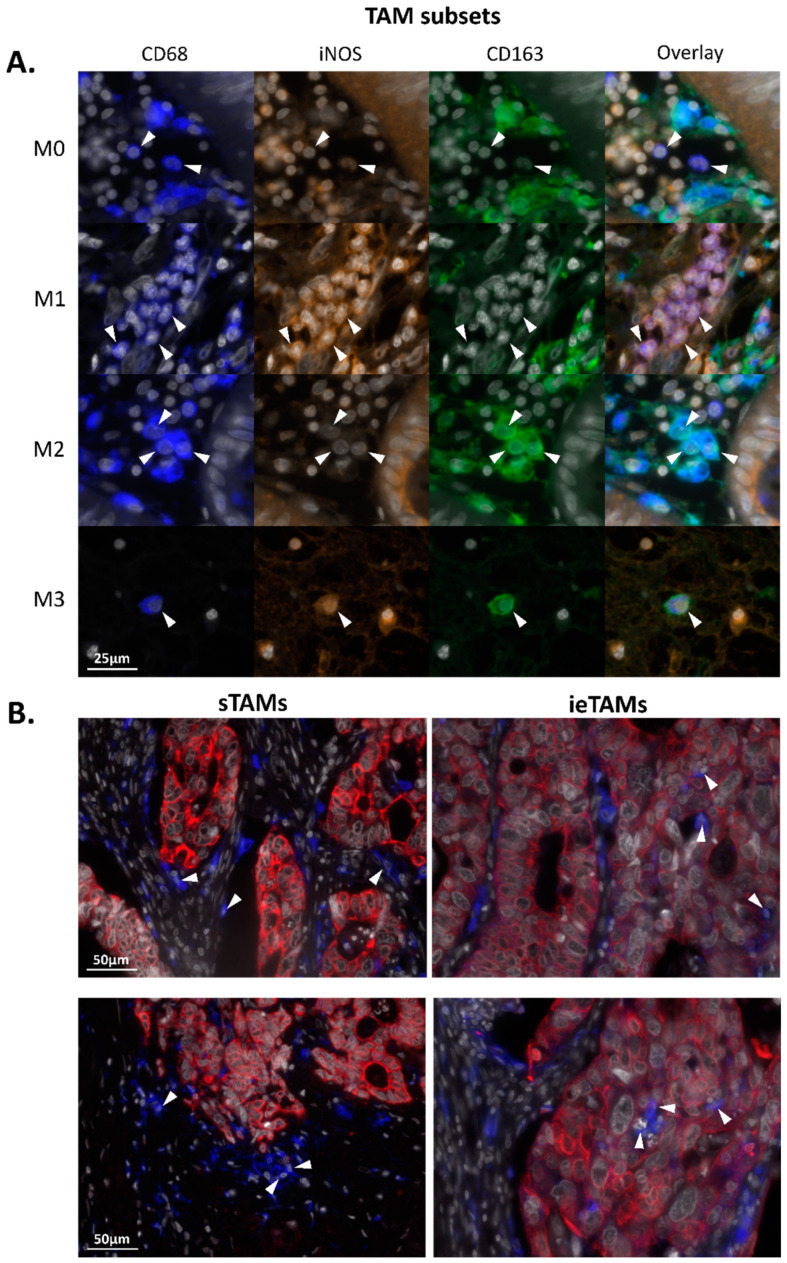
TAM subsets in primary colorectal tumors as visualized by multiplex immunofluorescence. (**A**) Image of M0 (CD68^+^iNOS^−^CD163^−^), M1 (CD68^+^iNOS^+^CD163^−^), M2 (CD68^+^iNOS^−^CD163^+^) and M3 (CD68^+^iNOS^+^CD163^+^) TAMs. (**B**) Representative images of colorectal tumors with high numbers of stromal TAMs (sTAMs) and intraepithelial TAMs (ieTAMs) (white: DAPI; red: cytokeratin^+^ tumor epithelium; blue: CD68^+^ TAMs). The white arrows indicate examples of TAMs with indicated phenotypes (**A**) or localizations (**B**). Abbreviations: CRC (colorectal cancer), iNOS (inducible nitric oxide synthase), ieTAM (intraepithelial TAM), sTAM (stromal TAM), TAM (tumor-associated macrophage).

**Figure 5:**
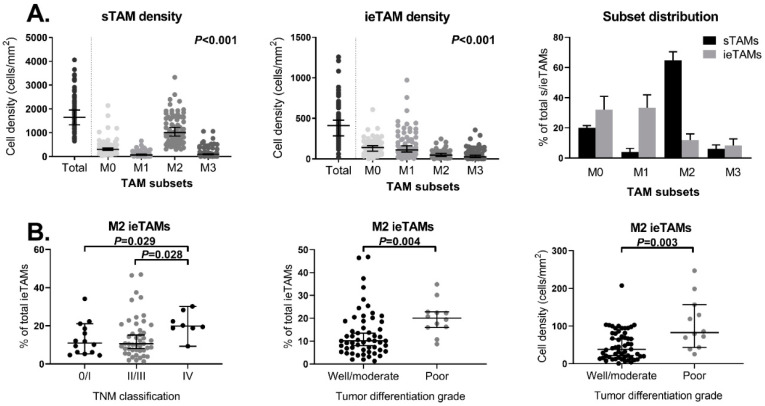
Distribution of the TAM subsets in the stromal and intraepithelial compartments of primary colorectal tumors as measured by multiplex immunofluorescence in relation to clinicopathological parameters. (**A**) Distribution of the sTAM and ieTAM subsets (CD68^+^iNOS^−^CD163^−^ M0, CD68^+^iNOS^+^CD163^−^ M1, CD68^+^iNOS^−^CD163^+^ M2 and CD68^+^iNOS^+^CD163^+^ M3 TAMs) in primary colorectal tumors (*N* = 72 and *N* = 68, respectively). (**B**) Associations between the percentage of M2 ieTAMs and TNM stage (stage 0/I, *N* = 14; stage II/III, *N* = 46; stage IV, *N* = 8), and between the percentage of M2 ieTAMs and M2 ieTAM density and tumor differentiation grade (well/moderate, *N* = 55; poor, *N* = 12) in primary colorectal tumors. The bars show the median with a 95% CI. Statistically significant *p*-values (≤0.05) are indicated in bold. Abbreviations: CI (confidence interval), CRC (colorectal cancer), iNOS (inducible nitric oxide synthase), ieTAM (intraepithelial TAM), sTAM (stromal TAM), TAM (tumor-associated macrophage), TNM (Tumor, Node, Metastasis).

Multiplex immunofluorescent imaging was used to identify the presence of stromal TAM (sTAM) and intraepithelial TAM (ieTAM) subsets in the primary tumors of CRC patients (*N* = 72). The sTAMs were successfully quantified in all 72 included primary colorectal tumors. Due to the occasional expression of CD68 in tumor epithelium cells, the total number of ieTAMs was sometimes overestimated. In total, four patients were identified as outliers with an overestimated cell density of the total ieTAMs and were therefore excluded from the analyses. M0 (iNOS^−^CD163^−^), M1 (iNOS^+^CD163^−^), M2 (iNOS^−^CD163^+^), and M3 (iNOS^+^CD163^+^) TAMs could be identified in primary colorectal tumors as illustrated in Figure 4A. Although the majority of TAMs were identified in the stromal compartment, TAMs were also observed to infiltrate the tumor epithelium compartment of some tumors. Figure 4B shows representative examples of colorectal tumors with high numbers of sTAMs and ieTAMs, respectively. In the majority of the studied tumors, the cell density of sTAMs was higher compared to ieTAMs (Figure 5A). In both stromal and epithelial tissue compartments, the TAM subsets were not equally distributed (*p* < 0.001, Figure 5A). The majority of sTAMs showed a M2-polarized phenotype (63% ± 17), whereas smaller numbers of M0 (21% ± 14), M1 (7% ± 8) and M3 (9% ± 9) sTAMs were found (Figure 5A). Interestingly, the distribution of ieTAMs showed a different pattern with relatively high numbers of M0 (37% ± 20) and M1 (36% ± 19) ieTAMs compared to low numbers of M2 (15% ± 10) and M3 (12% ± 12) ieTAMs (Figure 5A). Hence, sTAMs primarily showed an immunosuppressive (M2) phenotype whereas the majority of ieTAMs showed a naïve (M0) or inflammatory (M1) phenotype.

### 2.8. Increased M2 TAM Percentage in the Epithelial Compartment of Advanced Tumors

Next, the association between the cell density and distribution of different TAM subsets in the stromal (Table S1D,E) and epithelial (Table S1F,G) compartments of colorectal tumors and tumor characteristics was studied. The cell density and subset distribution of sTAMs were not associated with tumor characteristics (Table S1D and S1E, respectively). Although no correlation was observed between the percentage of M2 ieTAMs and the TNM stage (*p* = 0.205), an intergroup analysis revealed that patients with TNM stage IV tumors showed a higher percentage of M2 ieTAMs compared to TNM stage 0/I patients (*p* = 0.029) and TNM stage II/III patients (*p* = 0.028, Figure 5B). Furthermore, the percentage of M2 ieTAMs as well as the M2 ieTAM density were observed to be higher in patients with poorly differentiated tumors (N = 12) compared to patients with well or moderately differentiated tumors (*N* = 55, *p* = 0.004 and *p* = 0.003, respectively, Figure 5B). The cell density and subset distribution of the sTAMs and ieTAMs were not correlated with clinical outcome (data not shown). Interestingly, a trend was observed towards a positive correlation between the cell density of M2 sTAMs and the total percentage of circulating Tregs (*p* = 0.077), as well as for the cell density of M2 ieTAMs and Tregs (*p* = 0.103, Figure S4). No significant correlation was observed between the M2 sTAM or ieTAM density and serum sCD163 levels in CRC patients (*p* = 0.989 and *p* = 0.498, respectively, Figure S5).

## 3. Discussion

Innate immune cells in the circulation and tumor microenvironment participate in several stages of cancer progression [32]. The macrophage-associated molecule CD163 has been reported to be a prognostic biomarker in different cancer types [13,14,15,16,17,20,21,22], but its role in CRC is still unclear. We decided to study CD163 in a broader context, comprising both the tumor microenvironment and circulation of CRC patients. We investigated the CD163 expressed by circulating monocytes and TAMs, and its soluble circulating form (sCD163) in relation to clinicopathological parameters in CRC.

We evaluated the levels of sCD163 in serum and observed no differences between healthy donors and CRC patients, which is in agreement with studies on ovarian cancer [13], melanoma [14], and multiple myeloma [17], but in contrast with a study on hepatocellular carcinoma [15] and another study in CRC patients by Ding et al. [16]. The latter showed increased sCD163 levels in the total CRC patient population compared to healthy donors [16]. However, subset analyses revealed that early stage CRC patients showed comparable sCD163 levels with healthy donors [16]. This is in line with our results that showed increased sCD163 levels in CRC patients with advanced cancer stages. The total study population by Ding et al. contained relatively more patients with advanced cancer stages compared to our cohort, which may explain the discrepancy. Additionally, although the majority of our healthy donors had sCD163 levels within the reference range (0.7–3.9 mg/L), four healthy donors showed elevated levels. The healthy serum donors included in our study were spouses from cancer patients which may have been less healthy (i.e., relatively higher sCD163 levels) compared to the healthy donors included in the study by Ding et al. Excluding the healthy donor outliers from analysis did not affect the results of our analyses. In the present study, we observed no change in the sCD163 level of CRC patients after tumor resection. The post-operative serum samples were obtained within a range of 2–14 months after surgery, suggesting that resection of the primary tumor did not influence sCD163 levels or, alternatively, that it takes longer than the studied interval to detect a change in sCD163 levels after surgical resection of the tumor. In line with studies on other cancers [13,14,15,17] and a study on CRC specifically [16], a high sCD163 level was an independent predictor of DFS in our CRC cohort. Therefore, sCD163 levels may be used in a clinical setting as a prognostic biomarker. Additional research in a prospective setting is required to further investigate the potential use of sCD163 as prognostic biomarker in CRC.

We also observed comparable monocyte percentages and distribution of circulating monocyte subsets between CRC patients and healthy donors. Within the patient population, patients with the highest total monocyte frequency had more advanced tumors, represented by a high TNM classification, poor differentiation grade, and tumor-positive lymph nodes. This is in agreement with the meta-analysis by Tan et al. that reported an association between decreased lymphocyte-to-monocyte ratios and high tumor invasion depths and larger tumor sizes [7]. Additionally, in line with other studies [7,8], we observed a trend towards a shorter OS in CRC patients with a high percentage of total circulating monocytes. Such an association was not observed in our study for DFS. This may be due to the limited number of patients in our study cohort, but may also suggest that the monocyte percentages are associated with comorbidities instead of cancer-specific deaths. This has also been suggested by other studies that showed an association between high monocyte percentages and cardiovascular disease [33] and infectious diseases [34]. Furthermore, we showed in this study that the percentage of circulating monocytes positively correlated with the percentage of circulating Tregs. This is in line with other studies that suggested a positive feedback loop between these cell types [35,36]. Among others, monocytes can produce CCL5 and express the TIE2 receptor that recruits and expands immunosuppressive Tregs, respectively [37,38]. Additionally, Tregs produce IL-4, IL-10, and IL-13 that direct the differentiation of classical monocytes into M2 TAMs with further immunosuppressive functions [35,36]. In line with these studies, we observed a trend towards a positive correlation between the cell density of M2 TAMs and total percentage of circulating Tregs in CRC patients. Furthermore, we [39] and others [40,41,42] reported an association between a high percentage of circulating Tregs and poor clinical outcome in CRC patients. This suggests that monocytes/TAMs and Tregs closely interact and, additionally, play important roles in cancer development and progression.

The CD163 expression level was significantly lower on monocytes from CRC patients compared to healthy donors, suggesting that colorectal tumors influence the phenotype of monocytes. The mechanism behind the phenotypic regulation of monocytes, and other circulating innate immune cells, such as natural killer and natural killer T cells, are not fully understood, but immunosuppressive cytokines are thought to play a major role, along with the hypoxic conditions generated in the tumor microenvironment [39,43,44]. In our study, a decreased expression of CD163 on monocytes in CRC patients, compared to healthy donors, was not accompanied by elevated sCD163 levels. Additionally, there was no correlation between the CD163 expression on monocytes and sCD163 levels. This is in contrast with other studies that reported an inverse relation between the sCD163 and monocytic membrane expression of CD163 [11,45]. However, it is also argued that most sCD163 does not come from monocytes but from tissue macrophages [12,46], and thus, an increased shedding of CD163 from monocytes might not significantly increase the total sCD163. This hypothesis was further supported by Sugaya et al. who reported an association between the high infiltration of CD163^+^ cells in the skin and high sCD163 levels in patients with cutaneous T cell lymphoma [47]. These observations were not confirmed in our study. Importantly, other factors should also be taken into account when studying the shedding of the CD163 receptor from macrophages and monocytes, such as the presence and function of the ADAM17/TACE protease which mediates the cleavage of the CD163 receptor [11]. In the present study, the CD163 expression on circulating monocytes was not associated with tumor characteristics and clinical outcome.

We also characterized TAM subsets in primary colorectal tumors with an automated image analysis. Although the markers used in this study are often used to identify total (CD68), M1 (iNOS) and M2 (CD163) TAMs, we have to consider that TAMs show a remarkable functional plasticity and often express markers characteristic of both activation states [48]. Hence, the vast majority of macrophages have a functional phenotype on a scale in which M1 and M2 macrophages represent the extremes [49]. This makes subdividing TAMs into different subsets based on any type of cell surface markers with immunofluorescence difficult. In the present study, we observed that M2 TAMs were predominant in stromal tissue of colorectal tumors as also reported by others [27,50]. Additionally, Edin et al. reported more M2 compared to M1 TAMs at the invasive front of colorectal tumors [25]. This suggests that most sTAMs have an M2-polarized phenotype associated with immunosuppression. In contrast, Koelzer et al. reported that 40% of the total TAM population in colorectal tumors showed a M2 phenotype, whereas 60% presented an M1 phenotype [31]. A case-by-case analysis showed a positive correlation between the stromal CD68 counts and total tumor CD163 counts, but not with the total tumor iNOS counts [31]. Although not further studied, these results suggested that the majority of sTAMs had an M2-polarized phenotype. Since the total TAM population showed higher percentages of M1 TAMs compared to M2 TAMs, this might suggest that many ieTAMs with M1-polarized phenotypes were present in the colorectal tumors included in the study of Koelzer et al. In agreement with this observation, we reported an M0- and M1-biased TAM phenotype in the epithelial compartment of colorectal tumors in the present study. This observation is also supported by Kim et al. who reported low densities of M2 ieTAMs compared to total ieTAMs, suggesting that most ieTAMs had a phenotype other than M2, most likely M1 [50]. In summary, we and others showed that, in contrast to sTAMs, primary ieTAMs have an M1 phenotype that are associated with pro-inflammatory functions. As the function of TAMs might be dependent on their spatial distribution, it seems crucial to characterize TAMs in tumor epithelium and stroma separately in future studies.

To our knowledge, we are the first to study the presence of TAMs with M3 characteristics in human tissue. M3 TAMs have already been described in mice, also known as TAMs with an M1/M2 or M2/M1 switch phenotype, and thus are positive for both M1 (Ly6C) and M2 (CXCR1) markers [23]. Additionally, TAMs have also been reported to express both M1 and M2 markers in humans [24,25], although they have never been quantified and related to clinical parameters until now. In our study, we used a combination of the human M1 (iNOS) and M2 (CD163) markers to identify M3 macrophages (iNOS^+^CD163^+^). M3 TAMs are reported to have anti-tumor activities in an Ehrlich ascites [51] and a prostate cancer mouse model [52]. In our cohort, we did not observe an association between M3 TAM densities in the stroma or epithelium from primary colorectal tumors and tumor characteristics or clinical outcome. Therefore, the function of M3 TAMs in CRC remains unclear.

In the present study, the percentage of M2 ieTAMs positively correlated with the TNM stage and differentiation grade. Hence, M2 ieTAMs were more present in colorectal tumors with advanced stages. Two other studies reported the correlation between ieTAMs and tumor characteristics [31,50]. Whereas Koelzer et al. did not show any correlation between the total ieTAM counts and TNM stage or differentiation grade [31], Kim et al. showed higher total ieTAM counts in tumors with higher TNM stages [50]. In contrast, the M2 ieTAM counts were not associated with the TNM stage [50]. Additionally, we did not observe any correlations between the sTAM or ieTAM densities or subset distributions and clinical outcome of CRC patients in the present study. Koelzer et al. reported that, independent from TAM localization, high CD68 counts were associated with a longer OS [31]. In contrast, Kim et al. reported an association between a high intraepithelial CD68 density and worse outcome, but not for a high stromal CD68 density [50]. In summary, many contradictory findings have been reported on the association of TAMs in colorectal tumors with tumor characteristics and clinical outcome. Therefore, the role of TAMs in CRC remains elusive. Major issues in TAM research include the differences in used markers, techniques, and analyzing methods, which makes it difficult to compare studies, calling for more standardized assays. Based on our findings and others, it seems crucial to take the spatial distribution of TAMs in CRC into account. This should be investigated and validated in future studies.

In conclusion, we have shown that monocytes and sCD163 in the circulation are potential prognostic biomarkers to predict disease progression in CRC patients, whereas the TAM densities and phenotypes in the primary tumor are not, thereby emphasizing the importance of the innate systemic immune system in CRC disease progression.

## 4. Materials and Methods

### 4.1. Study Population and Patient-Derived Material

Seventy-eight patients diagnosed with tumor node metastasis (TNM) stage 0–IV CRC between 2001 and 2007 at Leiden University Medical Center (LUMC, the Netherlands) were included in the present study, and all underwent surgical resection. None of the patients received pre-operative chemotherapy nor were they diagnosed with Lynch syndrome. The pre-operative sera and peripheral blood mononuclear cells (PBMCs) were collected within a month prior to surgery. The post-operative serum samples were collected during routine checks in the outpatient clinic (mean 6.2 months after surgery, range 2–14). The post-operative samples obtained ≤2 months after surgery, or ≤5 months after the final therapy date in case a patient started adjuvant chemotherapy, were excluded as treatment may have influenced the peripheral blood immune system. Forty serum samples from healthy spouses of cancer patients and 10 PBMC samples from healthy blood donors were included as controls in this study. For the collection of serum samples, the peripheral blood of CRC patients was obtained (Dept. of Surgery, LUMC, The Netherlands) in BD Vacutainer serum separation transport tubes (BD Biosciences, Breda, The Netherlands). The tubes were centrifuged for 12 min at 1000× *g* after which the serum (supernatant) was frozen at −80 °C. The PBMCs were isolated and cryopreserved as described previously [39]. Formalin-fixed paraffin-embedded (FFPE) tumor tissue was obtained from primary CRC tissues (Dept. of Pathology, LUMC, The Netherlands). The clinicopathological data of all patients and healthy donors were available. All materials were obtained after approval by the Medical Ethical Committee of LUMC (protocol number P000.193). Written informed consent was obtained from all CRC patients and healthy donors included in the study.

### 4.2. Enzyme-Linked Immunosorbent Assay for the Detection of the sCD163 Levels in Serum

Serum samples were thawed and the sCD163 concentrations in serum were measured by an enzyme-linked immunosorbent assay (ELISA) using a BEP-2000 ELISA-analyzer (Dade Behring, Siemens, Erlangen, Germany) essentially as previously described [53]. Briefly, 96-wells plates were coated with polyclonal rabbit anti-CD163 IgG [9] diluted in a carbonate buffer (20 mM, pH 9.6). The wells were then washed three times in PBS, and 100 μL serum (diluted 1:101 in PBS/0.2% bovine serum albumin (BSA, Sigma-Aldrich, St. Louis, MO, USA)) supplemented with 0.25% Tween20 (Merck, Søborg, Denmark) was added and incubated for 90 min. After washing the wells, monoclonal anti-CD163 (clone GHI/61, BD Biosciences, Franklin Lakes, NJ, USA) was added and incubated for 60 min. After washing, peroxidase-labelled antibodies (goat anti-mouse immunoglobulins, DAKO, Glostrup, Denmark) were added and incubated for 60 min. The wells were washed and TMB ONE (Kem-En-Tec Nordic, Taastrup, Denmark) was added and incubated for 3 min. Finally, H_3_PO_4_ (1 M in water) was added to the wells and the plate was read on a BEP-2000 ELISA-analyzer. The internal control samples and serum standards were included in each run.

### 4.3. Multiparameter Flow Cytometry for the Detection of CD163 on Circulating Monocyte Subsets

The PBMC samples were thawed and cells were counted using a NucleoCounter NC-250 (Chemometec, Allerod, Denmark). The cell concentration was adjusted to 10 million/mL and the PBMCs were blocked for 15–30 min at room temperature (RT) with 50 μg/mL human IgG (CSL Behring, Bern, Switzerland) to prevent nonspecific antibody binding [54]. The PBMCs were then incubated with mouse anti-human antibodies against T cell and monocyte markers including CD3, CD4, CD8, CD14, CD16, CD25, CD45, CD127 and CD163 (for details see Table S2) as described previously [39]. Only one batch of each antibody type was used. Immediately after staining, the samples were analyzed on the LSRFortessa (BD Biosciences) flow cytometer running FACSDiva^TM^ software version 8.0 (BD Biosciences). FlowJo software version 10.1 (Tree Star Inc., Ashland, OR, USA) was used to analyze the data. In order to identify any inter-experimental variation, a buffy coat from a healthy donor obtained from Aarhus University Hospital, Denmark, was used as an internal control (PBMC reference sample). The threshold for positive staining was determined using unstained or fluorescence minus one (FMO) controls. In the present study, we used an FMO control for CD16. A standardized gating strategy based on the measurements of the PBMC reference sample was used to identify monocyte subpopulations (Figure S1). The expression of CD163 was then determined by the median fluorescence intensity (MFI) of the total monocyte population, as well as for the classical (CD14^++^CD16^−^), intermediate (CD14^++^CD16^+^), and nonclassical (CD14^+^CD16^++^) monocyte subsets separately. Additionally, regulatory T cells (Tregs, CD127^low^CD25^+^) were identified as described previously [39].

### 4.4. Multiplex Immunofluorescence for the Detection of TAMs

In total, 4 µm FFPE whole tumor tissue sections were cut and stained with macrophage-related markers using the Akoya Biosciences tyrosine amplification (TSA) method for multiplex immunofluorescence. Briefly, FFPE tissue sections were deparaffinized and rehydrated, and fixed with PBS/1% formaldehyde (Klinipath, Breda, The Netherlands) for 5 min at RT. Thereafter, the endogenous peroxidase activity was blocked by an incubation with 0.3% H_2_O_2_ (Millipore BV, The Netherlands) followed by a heat-induced antigen retrieval using a PT link module (DAKO). The tissue sections then underwent four staining cycles. Briefly, during every staining cycle, the sections were incubated with one type of primary antibody, anti-CD68 (KP1, DAKO), anti-iNOS (ab3523, AbCam, Cambridge, UK), anti-CD163 (NCL-L-CD163, DAKO), and finally anti-cytokeratin (EA1/EA3, DAKO). After each incubation round with primary antibodies, sections were incubated with horseradish peroxidase (HRP)-conjugated secondary antibodies (anti-mouse Envision, DAKO or anti-rabbit Envision, DAKO, depending on the species of which the primary antibodies were derived). The sections were then developed using Opal 570, Opal 690, Opal 520, or Opal 620 fluorophores (all from Akoya Biosciences) dissolved in 1x amplification buffer (Akoya Biosciences). After this visualization step, the sections were microwaved in AR6 buffer (Akoya Biosciences) to strip the antibody complexes from the sections and to perform antigen retrieval for the next staining cycle. The staining procedure described above was repeated three more times until all the epitopes of interest were targeted. Next, all sections were counterstained with DAPI (Sigma-Aldrich) and mounted with ProLong Gold Antifade Mountant (Thermo Fisher Scientific, Bleiswijk, The Netherlands).

### 4.5. Automated Image Analyses

The VECTRA 3.0 automated quantitative pathology imaging system (Akoya Biosciences) was used for imaging of the multiplexed-stained slides. The whole tissue sections were scanned at a 10× magnification. PhenoChart software (Akoya Biosciences, 1.0.4.) was used to randomly select 6 multispectral imaging (MSI) fields within the tumor regions, defined as areas containing at least 30% tumor epithelium based on the anti-cytokeratin staining and DAPI signal, which were then scanned at a higher resolution (20×). InForm software (Akoya Biosciences, 2.2.1) was used to prepare a spectral library of every fluorophore. Spectral unmixing was then performed on the multiplexed-stained slides and the background signals were extracted using InForm software. Thereafter, a tissue segmentation algorithm was trained using InForm software in order to automatically define tumor epithelium, stroma, and areas without tissue based on anti-cytokeratin antibodies and DAPI signals. A cell segmentation algorithm was set up based on the detection of cell nuclei using the DAPI signal. A phenotyping algorithm was trained to distinguish macrophages (CD68^+^) from non-macrophages (CD68^−^) within the tumor epithelium and stromal compartments separately. Finally, the iNOS and CD163 expression were scored on the identified TAMs using a set threshold to identify M0 (iNOS^−^CD163^−^), M1 (iNOS^+^CD163^−^), M2 (iNOS^−^CD163^+^), and M3 (iNOS^+^CD163^+^) TAMs. Subsequently, the cell density (cells/mm^2^) and subset distribution (percentage of the total stromal or intraepithelial TAMs) were calculated.

### 4.6. Statistical Analyses

Statistical analyses were performed using SPSS software (IBM SPSS Statistics 22, Chicago, IL, USA). Independent samples T tests and Mann–Whitney U tests were used in order to compare the markers between CRC patients and healthy donors. Dependent samples T tests were used to study the change in sCD163 concentrations between pre-operative and post-operative serum samples. Independent samples T tests, Mann–Whitney U tests, Kruskal–Wallis tests, ANOVA, and the Spearman’s rho correlation test were used to relate monocytes, sCD163, and macrophages with tumor characteristics. The Spearman’s rho test was used to study the correlation between the serum sCD163 levels and the CD163 expression on monocytes and TAMs. In addition, Kaplan–Meier analyses and log-rank tests were used to correlate monocytes, sCD163, and macrophages with patients’ OS and DFS. The OS was defined as the time from surgery until death, or the end of follow-up (censored). The DFS was defined as the time from surgery until the first sign of disease recurrence or until death, whichever came first, or the end of the follow-up (censored). A Cox regression analysis was used for the univariate and multivariate analyses. *p*-values ≤ 0.05 were considered statistically significant.

## Figures and Tables

**Table 1:** Clinicopathological characteristics of patients with CRC and healthy donors in the study. Statistically significant *p*-values (≤0.05) are indicated in bold. Abbreviations: CRC (colorectal cancer), PBMC (peripheral blood mononuclear cells), TNM (tumor, node, metastasis).

	CRC Patients	Healthy Serum Donors		Healthy PBMC Donors	
	(*N* = 78)	(*N* = 40)	*p*-Value	(*N* = 10)	*p*-Value
Age *			0.392		**0.028**
*Mean* (*years*)	65.9	63.8		48.8	
*Range* (*years*)	25–85	26–82		22–78	
Sex			0.597		0.951
*Female*	35 (44.9%)	20 (50.0%)		5 (50.0%)	
*Male*	34 (55.1%)	20 (50.0%)		5 (50.0%)	
Tumor location					
*Colon*	64 (82.1%)				
*Rectum*	14 (17.9%)				
TNM classification					
*Stage 0*	4 (5.1%)				
*Stage I*	12 (15.4%)				
*Stage II*	26 (33.3%)				
*Stage III*	26 (33.3%)				
*Stage IV*	10 (12.8%)				
Tumor differentiation					
*Well/moderate*	62 (79.5%)				
*Poor*	13 (16.7%)				
*Unknown*	3 (3.8%)				
Tumor-positive lymph nodes					
*No*	45 (57.7%)				
*Yes*	32 (41.0%)				
*Unknown*	1 (1.3%)				
Neoadjuvant radiotherapy					
*No*	69 (88.5%)				
*Yes*	9 (11.5%)				
Adjuvant chemotherapy					
*No*	49 (62.8%)				
*Yes*	29 (37.2%)				

* Age at time of surgery was used for patients and time of serum/PBMC donation for healthy donors.

**Table 2:** Univariate and multivariate analyses of sCD163 serum levels for the DFS of CRC patients. Univariate and multivariate analyses for DFS were generated for stage 0–III CRC patients (*N* = 58). The median sCD163 level (2.0 mg/L) was used as a cutoff. Statistically significant *p*-values (≤0.05) are indicated in bold. Abbreviations: CI (confidence interval), CRC (colorectal cancer), DFS (disease-free survival), HR (hazard ratio), sCD163 (soluble CD163), TNM (tumor, node, metastasis).

	Univariate Analysis for DFS			Multivariate Analysis * for DFS		
Parameter	HR	95% CI	*p*-Value	HR	95% CI	*p*-Value
Age (continuous)	1.0	1.0–1.1	0.340			
Age						
*≤70 years*	1.0					
*>70 years*	2.0	0.9–4.3	0.072			
Sex						
*Female*	1.0					
*Male*	1.4	0.7–3.1	0.358			
TNM classification						
*Stage 0/I*	1.0					
*Stage II*	2.0	0.5–7.6	0.299			
*Stage III*	5.9	1.7–20.4	**0.005**			
Tumor location						
*Colon*	1.0					
*Rectum*	1.9	0.8–4.5	0.117			
Tumor differentiation grade						
*Well/moderate*	1.0					
*Poor*	0.9	0.3–2.5	0.775			
sCD163 (continuous)	1.1	0.8–1.6	0.446	1.0	0.7–1.4	0.903
sCD163						
*Below-median*	1.0			1.0		
*Above-median*	3.1	1.4–7.1	**0.007**	2.4	1.0–5.7	**0.049**

* Corrected for age (categorized as ≤70 or >70 years age) and TNM classification.

**Table 3:** Univariate and multivariate analyses of sCD163 serum levels for the OS of CRC patients Univariate and multivariate analyses for OS were generated for stage 0–IV CRC patients (*N* = 64). The median sCD163 level (2.0 mg/L) was used as a cutoff. Statistically significant *p*-values (≤0.05) are indicated in bold. Abbreviations: CI (confidence interval), CRC (colorectal cancer), HR (hazard ratio), OS (overall survival), sCD163 (soluble CD163), TNM (tumor, node, metastasis).

	Univariate Analysis for OS			Multivariate Analysis * for OS		
Parameter	HR	95% CI	*p*-Value	HR	95% CI	*p*-Value
Age (continuous)	1.0	1.0–1.1	**0.039**			
Age						
*≤70 years*	1.0					
*>70 years*	2.9	1.4–6.1	**0.005**			
Sex						
*Female*	1.0					
*Male*	1.9	0.9–4.1	0.101			
TNM classification						
*Stage 0/I*	1.0					
*Stage II*	1.7	0.4–6.4	0.459			
*Stage III*	4.5	1.3–15.8	**0.018**			
*Stage IV*	30.7	6.6–143.0	**<0.001**			
Tumor location						
*Colon*	1.0					
*Rectum*	1.5	0.7–3.4	0.314			
Tumor differentiation grade						
*Well/moderate*	1.0					
*Poor*	1.5	0.6–3.4	0.393			
sCD163 (continuous)	1.2	0.9–1.6	0.303	1.0	0.7–1.4	0.960
sCD163						
*Below median*	1.0			1.0		
*Above median*	2.2	1.0–4.6	**0.040**	1.5	0.7–3.3	0.291

* Corrected for age (categorized as ≤70 or >70 years age) and TNM classification.

## Supplementary Materials

The following are available online at https://www.mdpi.com/1422-0067/21/16/5925/s1, Table S1: Association of monocyte percentages, sCD163 levels and TAM subsets with clinicopathological characteristics of CRC patients, Table S2: Flow cytometry antibody panel used for the identification of monocytes in peripheral blood of CRC patients, Figure S1: Flow cytometry gating strategy used for the identification of circulating monocyte subsets, Figure S2: Association between circulating monocytes and Tregs in CRC patients, Figure S3: Association between sCD163 levels and CD163 expression by monocytes in CRC patients, Figure S4: Association between circulating Tregs and M2 TAMs in CRC patients, Figure S5: Association between sCD163 levels and CD163 expression by TAMs in CRC patients.

## Abbreviations

CI	Confidence interval
CRC	Colorectal cancer
DFS	Disease-free survival
ELISA	Enzyme-linked immunosorbent assay
FFPE	Formalin-fixed paraffin-embedded
FMO	Fluorescence minus one
Hp-Hb	Haptoglobin-hemoglobin
HR	Hazard ratio
HRP	Horseradish peroxidase
iNOS	Inducible nitric oxide synthase
ieTAM	Intraepithelial TAM
sTAM	Stromal TAM
LUMC	Leiden University Medical Center
MFI	Median fluorescence intensity
MSI	Multispectral imaging
OS	Overall survival
PBMCs	Peripheral blood mononuclear cells
RT	Room temperature
sCD163	Soluble CD163
TAM	Tumor-associated macrophage
TNM	Tumor Node Metastases
Tregs	Regulatory T cells
TSA	Tyrosine amplification
