# Supplementary material for: CD163 as a Biomarker in Colorectal Cancer: The Expression on Circulating Monocytes and Tumor-Associated Macrophages, and the Soluble Form in the Blood
Source: Int J Mol Sci. 2020 Aug 18;21(16):5925. doi: 10.3390/ijms21165925 (PMC7460610; doi:10.3390/ijms21165925)
Supplement: Supplementary file 1 [file ijms-21-05925-s001.pdf]

Table S1: *Association of monocyte percentages, sCD163 levels and TAM subsets with clinicopathological characteristics of CRC patients.* **A.** sCD163 levels in serum. **B.** Subset distribution of circulating monocytes. **C.** Expression of CD163 on circulating monocyte subsets. **D.** Density of sTAMs in primary colorectal tumors. **E.** Subset distribution of sTAMs in primary colorectal tumors. **F.** Density of ieTAMs in primary colorectal tumors. **G.** Subset distribution of ieTAMs in primary colorectal tumors. Statistically significant *P*-values (≤0.05) are indicated in bold. Abbreviations: CRC (colorectal cancer), ieTAM (intraepithelial TAM), MFI (median fluorescence intensity), sCD163 (soluble CD163), SD (standard deviation), sTAM (stromal TAM), TAMs (tumor-associated macrophages), TNM (Tumor, Node, Metastasis).

**A.**		**sCD163 (mg/l)**		
**Parameters**	**No.**	**Mean**	**SD**	***P*-value**
TNM stage (Spearman)				0.141
TNM stage (ANOVA)				0.215
Stage 0/I	15	1.9	0.7	
Stage II/III	43	2.4	1.1	
Stage IV	6	2.7	1.1	
Tumor location				0.626
Colon	52	2.3	1.1	
Rectum	12	2.2	0.7	
Tumor differentiation grade				0.729
Well/moderate	48	2.3	1.0	
Poor	13	2.4	1.0	
Tumor lymph node invasion				0.355
Yes	26	2.4	1.2	
No	37	2.2	0.9	

**B.**		**Total monocytes** (% of CD45⁺ PBMCs)			**Classical monocytes** (% of total monocytes)			**Intermediate monocytes** (% of total monocytes)			**Nonclassical monocytes** (% of total monocytes)		
**Parameters**	**No.**	**Mean**	**SD**	***P*-value**	**Mean**	**SD**	***P*-value**	**Mean**	**SD**	***P*-value**	**Mean**	**SD**	***P*-value**
TNM stage (Spearman)				**0.004**			0.219			0.179			0.682
TNM stage (ANOVA)				0.060			0.215			0.165			0.641
Stage 0/I	14	23	13		89	6		3	7		4	2	
Stage II/III	25	28	12		88	6		5	6		4	2	
Stage IV	8	34	11		91	6		3	2		4	4	
Tumor location				0.874			0.874			0.961			0.826
Colon	35	28	14		89	7		4	6		4	3	
Rectum	12	26	8		89	4		4	4		4	2	
Tumor differentiation grade				0.055			**0.039**			0.948			0.886
Well/moderate	34	25	12		88	7		5	7		4	3	
Poor	11	34	15		92	3		2	1		4	2	
Tumor lymph node invasion				**0.011**			0.254			0.465			0.877
Yes	17	40	13		89	8		5	7		4	3	
No	30	24	11		89	5		4	5		4	2	

**C.**		**CD163 (MFI)** Total monocytes			**CD163 (MFI)** Classical monocytes			**CD163 (MFI)** Intermediate monocytes			**CD163 (MFI)** Nonclassical monocytes		
**Parameters**	**No.**	**Mean**	**SD**	***P*-value**	**Mean**	**SD**	***P*-value**	**Mean**	**SD**	***P*-value**	**Mean**	**SD**	***P*-value**
TNM stage (Spearman)				0.968			0.894			0.813			0.161
TNM stage (ANOVA)				0.837			0.896			0.485			0.286
Stage 0/I	14	10805	7656		11376	8560		17282	10209		4014	1949	
Stage II/III	25	1188	5021		12343	5491		17525	7839		3733	1976	
Stage IV	8	10912	4857		11571	5858		14009	3353		2817	1140	
Tumor location				0.762			0.806			0.542			0.188
Colon	35	11239	6000		11785	6772		16010	9411		3436	1778	
Rectum	12	11836	5370		12327	5754		19316	9639		4318	2030	
Tumor differentiation grade				0.704			0.700			0.969			0.277
Well/moderate	34	11366	6131		11915	6886		16375	8161		3760	1990	
Poor	11	10614	3749		11060	4192		17005	7109		3121	1449	
Tumor lymph node invasion				0.942			0.900			0.947			0.528
Yes	17	11308	4373		11763	5191		16151	4160		3372	1419	
No	30	11439	6534		12014	7178		17252	9644		3825	2079	

**D.**		**M0 sTAM density** (cells/mm^2)			**M1 sTAM density** (cells/mm^2)			**M2 sTAM density** (cells/mm^2)			**M3 sTAM density** (cells/mm^2)		
**Parameters**	**No.**	**Mean**	**SD**	***P*-value**	**Mean**	**SD**	***P*-value**	**Mean**	**SD**	***P*-value**	**Mean**	**SD**	***P*-value**
TNM stage (Spearman)				0.563			0.998			0.423			0.947
TNM stage (ANOVA)				0.876			0.621			0.239			0.835
Stage 0/I	14	407	520		93	97		1153	675		220	294	
Stage II/III	49	387	329		125	133		1092	621		174	225	
Stage IV	9	335	161		64	44		1418	618		143	131	
Tumor location				0.809			0.245			0.650			0.179
Colon	60	398	380		107	123		1166	655		154	190	
Rectum	12	316	179		134	111		1036	503		305	353	
Tumor differentiation grade				1.000			0.800			0.112			0.894
Well/moderate	58	400	384		117	127		1087	609		189	243	
Poor	13	336	187		94	83		1414	707		146	163	
Tumor lymph node invasion				0.553			1.000			0.802			0.508
Yes	30	391	322		107	104		1178	677		150	174	
No	42	379	381		114	132		1121	604		200	262	

**E.**		**M0 sTAMs** (% of total sTAMs)			**M1 sTAMs** (% of total sTAMs)			**M2 sTAMs** (% of total sTAMs)			**M3 sTAMs** (% of total sTAMs)		
**Parameters**	**No.**	**Mean**	**SD**	***P*-value**	**Mean**	**SD**	***P*-value**	**Mean**	**SD**	***P*-value**	**Mean**	**SD**	***P*-value**
TNM stage (Spearman)				0.952			0.970			0.419			0.731
TNM stage (ANOVA)				0.814			0.598			0.287			0.940
Stage 0/I	14	23	16		5	5		62	15		10	10	
Stage II/III	49	22	15		8	9		61	19		9	9	
Stage IV	9	17	5		4	3		71	8		8	5	
Tumor location				0.629			0.174			0.311			0.139
Colon	60	22	14		7	8		63	18		8	8	
Rectum	12	19	12		8	7		59	14		14	12	
Tumor differentiation grade				0.405			0.582			0.093			0.582
Well/moderate	58	23	15		7	8		61	18		10	10	
Poor	13	18	7		5	5		70	11		7	6	
Tumor lymph node invasion				0.615			0.973			0.900			0.398
Yes	30	21	12		7	7		63	17		9	9	
No	42	21	15		7	8		62	18		10	9	

**F.**		**M0 ieTAM density** (cells/mm^2)			**M1 ieTAM density** (cells/mm^2)			**M2 ieTAM density** (cells/mm^2)			**M3 ieTAM density** (cells/mm^2)		
**Parameters**	**No.**	**Mean**	**SD**	***P*-value**	**Mean**	**SD**	***P*-value**	**Mean**	**SD**	***P*-value**	**Mean**	**SD**	***P*-value**
TNM stage (Spearman)				0.743			0.293			0.901			0.092
TNM stage (ANOVA)				0.763			0.164			0.816			0.329
Stage 0/I	14	128	71		197	177		56	35		91	109	
Stage II/III	46	155	114		192	195		60	56		50	59	
Stage IV	8	132	80		73	41		61	34		26	23	
Tumor location				0.972			0.387			0.603			0.153
Colon	58	148	106		183	177		62	52		59	74	
Rectum	10	137	80		156	221		47	34		34	49	
Tumor differentiation grade				0.132			0.883			**0.003**			0.689
Well/moderate	55	137	90		186	193		49	40		54	71	
Poor	12	197	141		160	134		105	68		64	76	
Tumor lymph node invasion				0.740			0.684			0.586			0.176
Yes	27	146	84		178	203		53	39		41	50	
No	41	147	114		180	170		64	56		65	81	

**G.**		**M0 ieTAMs** (% of total ieTAMs)			**M1 ieTAMs** (% of total ieTAMs)			**M2 ieTAMs** (% of total ieTAMs)			**M3 ieTAMs** (% of total ieTAMs)		
**Parameters**	**No.**	**Mean**	**SD**	***P*-value**	**Mean**	**SD**	***P*-value**	**Mean**	**SD**	***P*-value**	**Mean**	**SD**	***P*-value**
TNM stage (Spearman)				0.076			0.430			0.205			0.137
TNM stage (ANOVA)				0.207			0.252			0.067			0.441
Stage 0/I	14	34	23		37	18		13	9		17	18	
Stage II/III	46	37	21		38	20		14	11		11	10	
Stage IV	8	45	12		26	9		21	6		9	9	
Tumor location				0.315			0.860			0.768			0.153
Colon	58	36	20		36	19		15	11		13	12	
Rectum	10	43	19		35	18		14	8		7	9	
Tumor differentiation grade				0.327			0.095			**0.004**			0.630
Well/moderate	55	36	21		38	20		13	10		12	12	
Poor	12	42	19		28	15		20	7		10	11	
Tumor lymph node invasion				0.151			0.739			0.826			0.205
Yes	27	41	20		35	20		14	8		10	11	
No	41	35	21		37	19		15	12		13	13	

Table S2: *Flow cytometry antibody panel used for the identification of monocytes in peripheral blood of CRC patients.* Abbreviations: AF (alexa fluor), APC (allophycocyanin), BV (brilliant violet), FITC (fluorescein isothiocyanate), nIR (near-infrared), PE (phycoerythrin), PE-Cy7 (phycoerythrin-cyanine7), PerCP (peridinin chlorophyll protein complex), V500 (violet500).

**Flow cytometry antibody panel**				
**Marker**	**Fluorochrome**	**Clone**	**Source**	**Staining concentration (µg/ml)**
CD3	BV605	SK7	BD Biosciences	1.25
CD4	PE-Cy7	SK3	BD Biosciences	0.03
CD8	AF700	RPA-T8	BD Biosciences	0.32
CD14	V500	Møp9	BD Biosciences	0.25
CD16	PerCP-eFluor710	3G8	eBioscience	0.75
CD25	APC	BC96	eBioscience	0.75
CD45	FITC	HI30	BD Biosciences	0.3
CD127	BV421	HIL-7R-M21	BD Biosciences	1.5
CD163	PE	Mac2-158	Trillium	0.5
Live/dead	nIR	-	Life Technologies	recommended

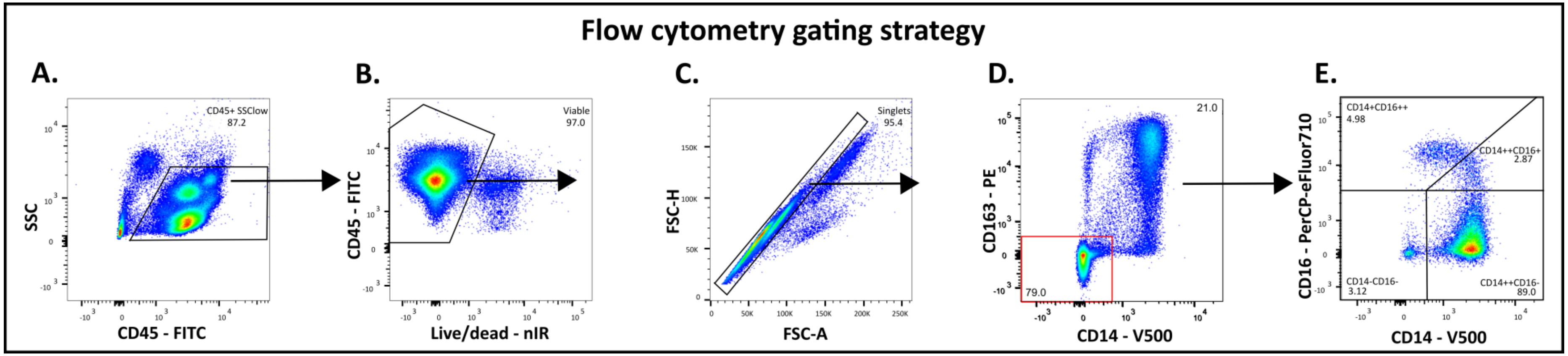

Figure S1: *Flow cytometry gating strategy used for the identification of circulating monocyte subsets.* **A.** Mononuclear cells (excluding CD45$^-$ cells and SSC^{high} cells). **B.** Viable mononuclear cells (excluding dead cells). **C.** Single mononuclear cells (excluding doublets). **D.** CD14$^+$ and/or CD163$^+$ monocytes (excluding CD14$^-$CD163$^-$ lymphocytes present in the red gate). **E.** Classical (CD14^{++}CD16$^-$), intermediate (CD14^{++}CD16$^+$) and nonclassical (CD14$^+$CD16^{++}) monocytes. Abbreviations: FSC (forward scatter), nIR (near-infrared), PBMC (peripheral blood mononuclear cells), SSC (side scatter).

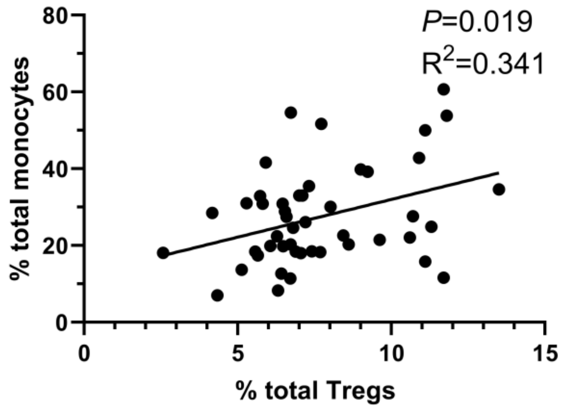

Figure S2: *Association between circulating monocytes and Tregs in CRC patients.* Correlation between the percentage of total circulating $CD14^+$ and/or $CD163^+$ monocytes and $CD127^{low}CD25^+$ Tregs in CRC patients (N=47). Abbreviations: CRC (colorectal cancer), Tregs (regulatory T cells).

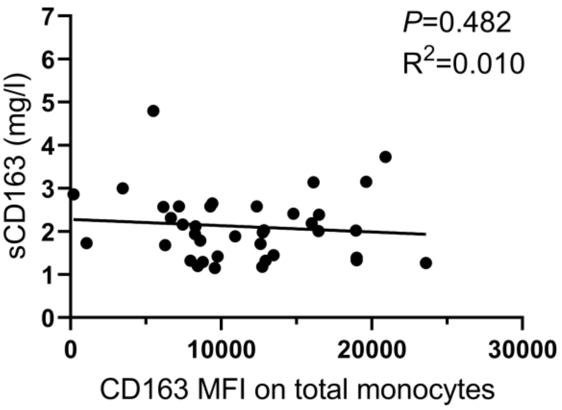

Figure S3: *Association between sCD163 levels and CD163 expression by monocytes in CRC patients*. Correlation between sCD163 levels and CD163 expression on circulating monocytes in CRC patients (N=38). Abbreviations: CRC (colorectal cancer), MFI (median fluorescence intensity).

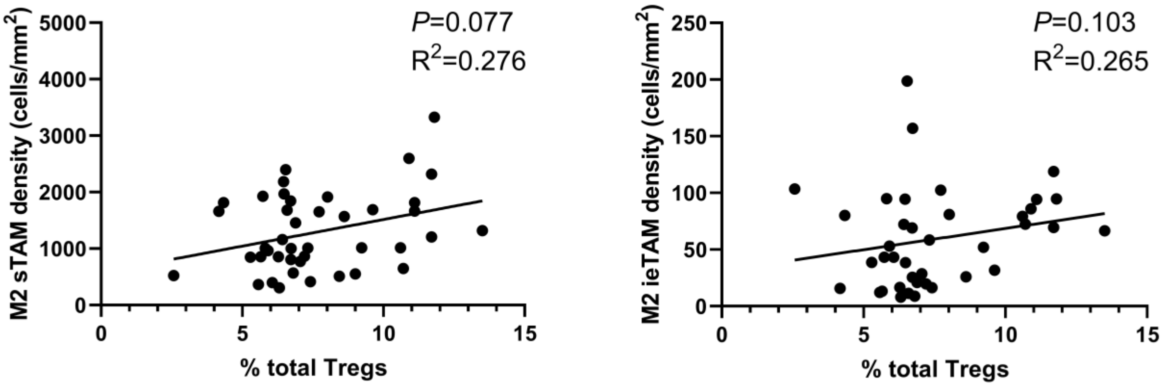

Figure S4: *Association between circulating Tregs and M2 TAMs in CRC patients.* Correlation between total circulating CD127^{low}CD25^{+} Tregs and CD68^{+}iNOS^{-}CD163^{+} M2 sTAM density (N=42) and ieTAM density (N=39) in CRC patients. Abbreviations: CRC (colorectal cancer), ieTAM (intraepithelial TAM), iNOS (inducible nitric oxide synthase), sTAM (stromal TAM), TAM (tumor-associated macrophage), Tregs (regulatory T cells).

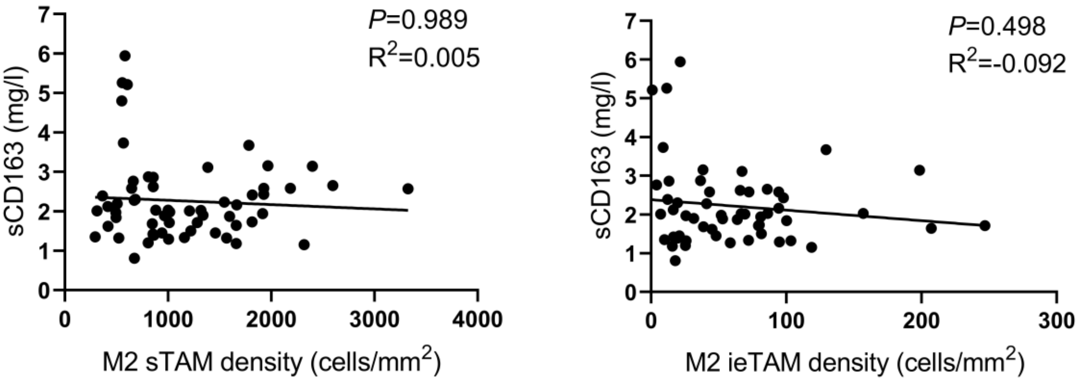

Figure S5: *Association between sCD163 levels and CD163 expression by TAMs in CRC patients*. Correlation between sCD163 levels and $CD68^{+}iNOS^{-}CD163^{+}$ M2 sTAM density (N=60) and ieTAM density (N=56) in CRC patients. Abbreviations: CRC (colorectal cancer), iNOS (inducible nitric oxide synthase), ieTAM (intraepithelial TAM), sTAM (stromal TAM), TAM (tumor-associated macrophage).
